# Characterization of Phenolic Profile and Biological Properties of *Astragalus membranaceus* Fisch. ex Bunge Commercial Samples

**DOI:** 10.3390/antiox13080993

**Published:** 2024-08-16

**Authors:** Saba Shahrivari-Baviloliaei, Ilkay Erdogan Orhan, Nurten Abaci Kaplan, Agnieszka Konopacka, Krzysztof Waleron, Alina Plenis, Agnieszka Viapiana

**Affiliations:** 1 Department of Analytical Chemistry, Medical University of Gdansk, Gen. J. Hallera 107, 80-416 Gdansk, Poland; saba.shahrivari@gumed.edu.pl; 2 Department of Pharmacognosy, Faculty of Pharmacy, Gazi University, 06330 Ankara, Türkiye; nurtenabaci@gazi.edu.tr (N.A.K.); ilkay.erdoganorhan@lokmanhekim.edu.tr (I.E.O.); 3 Department of Pharmacognosy, Faculty of Pharmacy, Lokman Hekim University, 06510 Ankara, Türkiye; 4 Department of Pharmaceutical Microbiology, Medical University of Gdansk, Gen. J. Hallera 107, 80-416 Gdansk, Poland; agnieszka.konopacka@gumed.edu.pl (A.K.); krzysztof.waleron@gumed.edu.pl (K.W.)

**Keywords:** *Astragalus membranaceus* Fisch. ex Bunge, phenolic composition, antioxidant activity, antimicrobial activity, enzyme inhibitory activity, acetylcholinesterase (AChE), butyrylcholinesterase (BChE), tyrosinase (TYR)

## Abstract

*Astragalus membranaceus* Fisch. ex Bunge (syn. *Astragalus mongholicus* Bunge) is one of the notable medicinal and food plants. Therefore, the aim of this study was to calculate the phenolic composition and antioxidant, antimicrobial, as well as enzyme inhibitory [acetylcholinesterase (AChE), butyrylcholinesterase (BChE), and tyrosinase (TYR)] activities with chemometric approaches of the hydromethanolic and water extracts of commercial *A. membranaceus* samples. Ten individual phenolic compounds were determined using high-performance liquid chromatography (HPLC), and only quercetin was found at a level of above 80 µg/g DW in both extracts. Moreover, the highest antioxidant activity in DPPH, FRAP, ABTS, and CUPRAC assays was found in the sample containing the roots in loose form from USA. *A. membranaceus* extracts displayed the inhibition zone diameters within the range from 10 to 22 mm antimicrobial activity against *S. aureus*, while there were no inhibition zones in any extracts in case of *E. coli*. The extracts of *A. membranaceous* showed an inhibition rate below 40% against TYR, and among tested extracts, only two samples were able to inhibit BChE with IC_50_ values of above 30 µg/mL. Correlation analysis showed a highly positive relationship between their phenolic composition and antioxidant activity. Concluding, the obtained results confirmed that *A. membranaceus* commercial samples could be an important dietary source of natural antioxidants.

## 1. Introduction

*Astragalus* L. (Fabaceae) belongs to the largest genus of vascular plants, comprising about 2900 species, which are distributed in America and most of the species in Eurasia [1,2]. Some of them growing in Asia are a source of gum tragacanth, an economically important natural product. Moreover, *Astragalus* L. species have been used for medicinal purposes for approximately 2000 years [3]. They have been traditionally used in folk medicine as an analgesic, anticancer, antidiarrheal, anti-infective, anti-inflammatory, antifatigue, antiperspirant, antianorexic, and stimulant, hypoglycemic, tonic, laxative, diuretic, and narcotic agent. In Iranian traditional medicine, the roots of *Astragalus* species are applied to treat a variety of ailments, including diabetes, respiratory infections, and leukemia [4]. Due to its medical potential as an immunostimulant and/or anticancer herbal medication, breakthroughs in the study of *Astragalus* sp. have been published in recent years.

*Astragalus membranaceus* Fisch. ex Bunge (syn. *Astragalus mongholicus* Bunge) (Fabaceae) is native to the northern and eastern parts of China, as well as Korea and Mongolia. It is widely distributed in temperate regions of Asia, Europe, and North America [5]. This plant has a long history of medicinal usage as a component of Chinese herbal formulation to treat patients with a variety of symptoms, including fatigue, chronic diarrhea, anorexia, and abnormal uterine bleeding [6,7]. *A. membranaceus* is valued for its root, known as Astragali radix, and is one of the major Traditional Chinese Medicinal (TCM) herbs used to boost immunity. It is often recommended during cancer and diabetes therapy [8]. It has also been utilized to treat skeletal muscular atrophy and tiredness syndrome [9]. Moreover, the plant possesses anti-inflammatory, antioxidant, immunomodulatory, anticancer, antitumor, antihyperglycemic, hypolipidemic, hepatoprotective, and diuretic properties [10,11]. In traditional medicine, *A. membranaceus* is mainly used as a tonic, often taking the form of common beverages such as teas and tinctures or as dietary supplements, while in the market, Astragalus root can be found as a capsule, liquid extract, or tea. Some recommend boiling the Astragalus directly into tea to release its active compounds [7,12,13].

The major bioactive components of *A. membranaceus* are saponins, polysaccharides, and flavonoids [14,15,16]. Saponins are a group of glycosides that include triterpenoids and steroids [17]. According to recent studies, saponins have been shown to possess antioxidant and anti-inflammatory properties, and they influence cognitive behavior. Astragalosides I, II, and IV, along with isoastragalosides I and II, account for approximately 80% of the total saponins present in Astragalus [18]. According to a study by Zhang et al. [19], astragaloside IV shows therapeutic effects in preventing neurodegenerative illnesses due to its anti-inflammatory, antioxidant, and antiapoptotic activities. Astragalus polysaccharide is a water-soluble heteropolysaccharide that has been utilized to slow the progression of Parkinson’s disease [20]. It has been shown to possess immune-enhancing, anti-inflammatory, and other pharmacological properties to successfully treat and ameliorate various nervous system-related disorders and nerve injury symptoms and to improve neurotrophic and neurological function [21]. Flavonoids include a group of both aglycone and glycone forms of calycosin, formononetin and ononin, of which calycosin and calycosin-7-O-β-D-glucoside belong to the major compounds related to bioactivity and are the recognized marker compounds for the chemical evaluation of A. membranaceus [22].

More than 200 chemical constituents have been isolated and identified from the root of *A. membranaceus* [23]. Li et al. [24] extracted and separated six active compounds, namely calycosin-7-O-β-d-glucoside, pratensein-7-O-β-d-glucoside, formononetin-7-O-β-d-glucoside, calycosin, genistein, and formononetin from the root of *A. membranaceus* and showed to be natural acetylcholinesterase inhibitors. Zhang et al. [25] isolated 24 secondary metabolites (isoflavonoids, astragalasides, and benzoquinone) from *A. membranaceus* roots and were shown to possess potent anti-inflammatory activity. However, many scholars have recently started to exploit the *A. membranaceus* stems and leaves as non-traditional medicine, and few studies have investigated the potential of their active components. Liu et al. [26] extracted and separated ten active components namely, wogonin, ononin, isoquercitrin, calycosin-7-glucoside, 3-hydroxy-9,10-dimethoxyptercarpan, hyperoside, 7,2′-dihydroxy-3′,4′-dimethoxyisoflavan, baicalein, calycosin, and soyasaponin from *A. membranaceus* stems and leaves, and the potential anti-Alzheimer’s disease effect of the obtained bioactive compounds was verified using molecular docking analysis. Liu et al. [27] extracted and evaluated flavonoid and triterpenoid distribution in roots, stems, leaves, petioles, and flowers of *A. membranaceus*, together with 13 of their metabolites, showing that isoliquiritigenin, liquiritigenin, daidzein, and bioactive isoflavones accumulate in both roots and flowers. Moreover, it also possesses components such as anthraquinones, alkaloids, amino acids (e.g., canavanine, proline, arginine, aspartic acid, asparagine, and alanine), β-sitosterol, and metallic elements (e.g., zinc, rubidium, iron, and manganese) [14].

Several studies have been conducted on the phenolic composition, antioxidant and antimicrobial activity, cholinesterase or tyrosinase inhibitory activity of extracts and single components of *A. membranaceus*. However, all of these studies are based on non-commercial (wild) *A. membranaceus*. Some studies have revealed differences in chemical composition between wild and commercial plant samples [28,29,30]. Commercial samples may yield completely different amounts of secondary metabolites and biological activity. These differences may be related to production and processing and also may include cultivation methods, harvest time and all post-harvest processes [22]. They may be related to processing (drying, preservation, packaging) and storage time. Commercial plant products nowadays are receiving worldwide attention for the prophylaxis of several diseases. In the case of *A. membranaceus*, the European Union has allowed its extracts to be marketed as novel food [31]. Because of this it is important to carefully analyze their quality. To our knowledge, there are no reports on the chemical composition and biological activity of commercial samples of *A. membranaceus*. For this reason, the present study aims to evaluate the phenolic composition and biological properties of commercial *A. membranaceus* samples. Cholinesterase enzymes are an important target for the treatment of several neuro-degenerative disorders. AChE inhibitors are currently the most prescribed drug class for the treatment of Alzheimer’s disease (AD) based on the treatment relevant to the cholinergic hypothesis [32,33,34,35,36]. Tyrosinase is a copper-containing enzyme that plays a multifaceted role in the pathology of Parkinson’s disease (PD) through its involvement in oxidative stress, neuromelanin formation, and inflammatory responses [37,38,39]. It is predominantly expressed in melanocytes, cells that produce melanin but is also found in the substantia nigra of the brain, a region significantly affected by PD. According to the few published studies *A. membranaceus* and its bioactive compounds have been found to be potent at treating neurodegenerative diseases and also a potential source of natural tyrosinase inhibitors. Therefore, in the current study, the extracts of the *A. membranaceus* commercial samples were tested for their acetylcholinesterase (AChE), butyrylcholinesterase (BChE), and tyrosinase (TYR) inhibitory effects using microtiter assays. The development of drug-resistant pathogens has evoked interest in screening natural resources in order to introduce novel antimicrobial substances. Moreover, the adverse effect of oxidative stress due to an excessive formation of free radicals is another serious issue that causes important destructive effects on human health. Nowadays, medicinal plants, as a natural source of antimicrobial and antioxidant constituents with limited side effects are attaining special interest. For this reason, the antimicrobial and antioxidant activities of the *A. membranaceus* commercial samples were investigated in the current study.

## 2. Materials and Methods

### 2.1. Sample Materials

Ten commercial samples (no. 1–10) of *A. membranaceus* were from a local supermarket (Auchan, Gdansk, Poland), herbal stores (Nagietek, Gdansk, Poland and Fragaria, Gdansk, Poland), and pharmacy stores (Dr Max, Gdansk, Poland and Gemini, Gdansk, Poland). All samples were stated to contain root tissue of the plant, wherein five samples were cut roots (no. 1, 3, 4, 6, and 7), two of them were in capsule form (2 and 5), and three of them were in powder form (8, 9, and 10). Five samples were products of China (no. 3, 4, 6, 8, and 9), two of them came from Poland (no. 1 and 2), two from the USA (no. 5 and 10), and one from Russia (no. 7). Moreover, samples no. 1, 2, and 5 were labeled as dietary supplements. The dry samples were pulverized in a water-cooled Knifetec 1095 grinder (Foss Tecator, Höganäs, Sweden) at 20 °C, and the samples were stored in a light-proof desiccator until further analysis.

### 2.2. Standards, Solutions, and Reagents

For the chemical analyses, the following high-purity standards (>98%), e.g., 2,2-azinobis(3-ethylbenzothiazoline-6-sulfonic acid) diammonium salt (ABTS reagent), 2,2-diphenyl-1-picrylhydrazyl (DPPH reagent), 4-chloro-7-nitrobenzofurazan (NBD-Cl) and ten analytical standards including gallic acid (GA), protocatechuic acid (PAT), vanillic acid (VA), *p*-coumaric acid (*p*CA), ferulic acid (FA), cinnamic acid (CNA), rutin (RUT), quercetin (Q), apigenin (API), and naringenin (NAR) were obtained from Sigma-Aldrich (St. Louis, MO, USA). Aluminum chloride (AlCl_3_) was purchased from Across Organics (Morris Plains, NJ, USA), while the rest of the reagents were from POCh (Gliwice, Poland). Redistilled water was prepared by triple distillation of water in a Destamat ^®^bi-18 system (HeraeusQuarzglas, Hanau, Germany).

The determination of total phenolic contents (TPCs), total flavonoid contents (TFCs), total phenolic acid contents (TPACs), and antioxidant activities of the samples was performed with a Metertech UV/Vis spectrophotometer (Nankang, Taipei, Taiwan) by determining the absorbance with 10 mm quartz cuvette at the wavelength described below in the corresponding sections [26].

### 2.3. Sample Preparation

In this work, hydromethanolic and water extracts (infusions) of *A. membranaceus* commercial samples were prepared. For the hydromethanolic extracts, 1.0 g of plant sample was sonicated with 4 mL of methanol-water mixture (80:20, *v/v*) for 20 min at 25 °C with the use of an ultrasonic bath (Emag, Salach, Germany). The suspension was centrifuged for 15 min at 8000 rpm (EBA-20S, Hettich, Tuttlingen, Germany), and the supernatant was transferred into a volumetric flask. This procedure was repeated twice, and the extracts were combined and diluted up to 20 mL with a methanol-water mixture (80:20, *v/v*).

For the water extracts (infusions), 1.0 g of plant material was infused with boiling distilled water (100 mL) for 15 min. After that, the infusion was filtered through a 0.25 μm nylon filter film (Mecherey, Nagel, Germany), and 20 μL of the filtrate was injected into the HPLC system.

### 2.4. Phytochemical Composition

#### 2.4.1. Chromatographic Conditions

Chromatographic separation of the selected phenolic compounds was carried out according to the protocol previously reported by Viapiana et al. [40]. The equipment used was a Merck-Hitachi LaChrome device (Darmstadt, Germany), and the detection and quantification of the compounds was based on the method described previously [40]. The detection wavelengths were set at 280 nm (GA, VA, PAT, CNA, and NAR), 320 nm (FA and *p*CA), and 370 nm (RUT, API, and Q). Identification of the phenolic compounds was performed by comparing the retention times of the detected compounds with those of commercial standards, as well as by spiking a sample with commercial standards. The method developed for the measurement of ten phenolic compounds was validated by linear range, the limit of detection (LOD), the limit of quantification (LOQ), precision, and recovery in accordance with the procedure described above [40].

#### 2.4.2. Total Phenolic Contents (TPCs)

The total phenolic contents in the samples were estimated according to the Folin–Ciocalteu method [41] with some modifications. In brief, a volume of 0.1 mL of each extract was mixed with 0.2 mL of Folin–Ciocalteu reagent. After 3 min, 0.2 mL of sodium carbonate (7%) was added. The tube content was well mixed and incubated for 60 min away from light, and the absorbance was read at 760 nm. The TPCs were quantified from the standard curve of gallic acid (10–70 μg/mL) and were expressed as µg of gallic acid equivalents (GAE) per gram of dry weight (µg GAE/g DW) for the hydromethanolic extracts, and as mg of gallic acid equivalents (GAE) per gram of dry weight (mg GAE/g DW) for water extracts of *A. membranaceus*.

#### 2.4.3. Total Flavonoid Contents (TFCs)

The total flavonoid contents in the hydromethanolic and water extracts were quantified as described by the European Pharmacopoeia [42]. A volume of 1 mL of each extract was mixed with 0.1 mL of 5% AlCl_3_ (*w/v*) and 1.4 mL of the acetic acid and methanol (1:19) mixture and then allowed to stand for 30 min. After that, the absorbance was measured at 425 nm. The TFCs were evaluated based on the quercetin standard (20–200 μg/mL), and results were expressed as µg of quercetin equivalents per gram of dry weight (µg QE/g DW).

#### 2.4.4. Total Phenolic Acid Contents (TPACs)

The total phenolic acid contents in the samples were estimated using Arnov’s reagent as described in the Polish Pharmacopoeia VI [43]. In brief, a volume of 1.4 mL of each extract was mixed with 0.2 mL chloric acid (0.5 N), 0.2 mL Arnov’s reagent and 0.2 mL sodium base (1 N). After that, the tube content was mixed, and the absorbance was measured at 490 nm. The TPACs were quantified from the standard curve of caffeic acid (5–30 μg/mL) and were expressed as µg of caffeic acid equivalents per gram of dry weight (µg CAE/g DW).

### 2.5. Activity Assays

#### 2.5.1. In Vitro Antioxidant Activities

The free radical DPPH scavenging activity of the *A. membranaceus* extracts was evaluated according to Tuberoso et al. [44]. In brief, 0.4 mL of each extract was added to 1.6 mL of freshly prepared DPPH methanolic solution (100 μmol/L). Next, the solution was well mixed and allowed to stand in the dark at room temperature for 10 min. After that, the absorbance was measured at 517 nm, and the results were expressed as mg of Trolox equivalents per gram of dry weight (mg TAE/g DW).

The ABTS assay was estimated using the method proposed by Arnao et al. [45] with some modifications. A volume of 0.05 mL of each extract was added to 2 mL of ABTS solution. After 6 min, the absorbance was measured at 734 nm, and the results were expressed in mg of Trolox equivalents per gram dry weight (mg TAE/g DW).

The FRAP assay was carried out using the method proposed by Benzie and Strain [46]. A volume of 0.05 mL of each extract was added to 2.25 mL of FRAP solution, and after 30 min, the absorbance was measured at 593 nm. The results were expressed in mg of ferrous ion equivalents per gram dry weight (mg Fe^2+^/g DW).

The CUPRAC assay was realized using the method of Apak et al. [47] with some modifications. A volume of 0.05 mL of each extract was added to 1 mL of neocuprine ethanolic solution (7.5 mM), 1 mL of copper chloride solution (0.01 M), and 1 mL of ammonium acetate buffer solution (pH: 7.00). After 30 min the absorbance was measured at 495 nm, and the results were expressed in mg ascorbic acid equivalents per gram dry weight (mg AAS/g DW).

#### 2.5.2. Antibacterial Activity Assay

A preliminary study of the antimicrobial activity of water extracts of *A. membranaceus* using an agar well diffusion test was carried out. Two reference strains of bacteria, i.e., *Staphylococcus aureus* ATCC 6538 and *Escherichia coli* ATCC 8739, were used. Molten-cooled MH agar (35 mL) was inoculated with 1 mL of a suspension of the appropriate bacterium at a density of 10^6^ CFU/mL, then poured into the sterile petri dish with cylinders set. Upon solidification of the agar, the cylinders were removed to wells with a diameter of 7 mm. Next, 0.3 mL of each extract (300 mg/mL) was added to respective wells. After the pre-incubation of 1 h at room temperature, the plates were incubated for 24 h at 37 °C to obtain bacterial growth. After incubation, the diameter of the growth inhibition zone was measured.

#### 2.5.3. Microtiter Assays for AChE/BChE and TYR Inhibition

##### ChE Inhibition

The inhibition of electric eel acetylcholinesterase (AChE, Type-VI-S, EC 3.1.1.7, Sigma, St. Louis, MO, USA) and horse serum butyrylcholinesterase (BChE, EC 3.1.1.8, Sigma, St. Louis, MO, USA) was assessed using a modified version of Ellman’s method [48]. The reaction mixture consisted of 140 µL of 0.1 mM sodium phosphate buffer (pH 8), 20 µL of 5,5′-dithio-bis(2-nitrobenzoic) acid (DTNB, 0.4 mM, Sigma, St. Louis, MO, USA). Finally, 20 µL of the enzyme (either AChE or BChE, 5.32 x 10^−3^ U) and 20 µL of the test samples were added in the mixture. This mixture was incubated for 15 min at 25 °C. Following incubation, 10 µL of acetylthiocholine iodide (0.4 mM) or butyrylthiocholine chloride (0.4 mM) was added. These substrates react with DTNB, producing a yellow-colored 5-thio-2-nitrobenzoate anion. The absorbance was measured at 417 nm using a VersaMax 96-well ELISA microplate reader (Molecular Devices, San Jose, CA, USA). Galanthamine served as the reference drug for comparison.

##### TYR Inhibition

The inhibition of TYR (EC 1.14.1.8.1, 30 U, mushroom tyrosinase, Sigma, St. Louis, MO, USA) was evaluated with L-DOPA as the substrate using a modified dopachrome method [49]. This assay was performed in a 96-well microplate. In brief, each well contained a sample aliquot dissolved in DMSO, combined with 80 μL of phosphate buffer (pH 6.8), 40 μL of TYR, and 40 μL of L-DOPA. The absorbance was measured at 475 nm using an ELISA microplate reader (VersaMax, Molecular Devices, San Jose, CA, USA). The results were compared against a control (DMSO), and α-kojic acid (Sigma, St. Louis, MO, USA) served as the reference.

##### Data Processing for Enzyme Inhibition Assays

The enzyme inhibition assays in this study were analyzed using Softmax PRO 4.3.2.LS software. The inhibition percentages of AChE, BChE, and TYR were calculated by comparing the reaction rates of the test samples to those of the control samples. The extent of the enzymatic reaction was determined using the following equation: I% = (C-T)/C × 100, where I% is the activity of the enzyme as percent inhibition. E value expresses the effect of the test sample or the positive control (reference inhibitor) on the enzyme activity of AChE/BChE/TYR. The inhibition was articulated as the percentage of the remaining activity in the presence of the test sample or positive control. C value expresses the absorbance of the control solvent (blank) in the presence of an enzyme. T is the absorbance of the tested sample or positive control/reference inhibitor in the solvent in the presence of the enzyme. Data are presented as average inhibition ± standard deviation (S.D.). The results were taken from at least three independent experiments implemented in triplicate.

### 2.6. Statistical Analysis

All samples were analyzed in triplicate. Statistical comparisons were performed using the one-way analysis of variance (ANOVA) test, followed by Tukey’s HSD. All the data was expressed as means values with the standard deviations (mean ± S.D.). The correlation between *A. membranaceus* extracts based on the chemical composition, antioxidant activity, and BChE/TYR inhibitory activity was calculated in a Pearson correlation analysis. Statistical data analysis was performed using Statistica 13.3 software (StatSoft Inc., Tulsa, OK, USA) on the basis of parametric tests with the level of significance of *p* < 0.05.

## 3. Results

### 3.1. Analysis of TPCs, TFCs, and TPACs

The results of TPCs, TFCs, and TPACs for hydromethanolic and water extracts of *A. membranaceus* commercial samples are shown in Table 1. The obtained results revealed that TPCs, TFCs and TPACs in the water extracts were significantly higher (*p* < 0.05) than those in the hydromethanolic extracts of the plant. In addition, the extracts of sample no. 5 (dietary supplement in the capsule form, from the USA) were the richest in TPCs, TFCs, and TPACs, while the extracts of sample no. 4 (cut roots from China) were the poorest among all analyzed *A. membranaceus* samples.

The available information on the phenolic contents of the studied *A. membranaceus* is very sparse. Sheng et al. [50] found higher TPCs and TFCs in extracts of *A. membranaceus* root from Mongolia, in the range from 135.23 to 197.40 mg GAE/g extract and 52.27 to 112.75 mg CE/g extract, respectively. Li et al. [51] found lower values of TPCs in Chinese fresh samples of *A. membranaceus*—from 12.29 to 108.42 mg chlorogenic acid equivalent/g dried extract, while values of TFCs detected in the range from 6.58 to 265.71 mg RUT/g dried extract. Gebalski et al. [52] also found TPCs and TFCs in *A. membranaceus* roots of Chinese origin at a level of 87.90 mg GAE/g of the sample and 20.90 mg QE/g of the sample, respectively. Moreover, they also detected TPACs at a higher level of 1.1 mg CAE/g of the sample than in this study. The TPCs, TFCs and TPACs obtained in this study differ from those in the relevant literature. The differences between the obtained data in this study and the literature data are due to the fact that the chemical composition of plants depends on many factors, such as plant species characteristics, collection time, morphological part of the plant, storage, soil type, agronomic practices, climatic factors and technological treatments as well as differences in the cultivars or varying stress conditions of the vegetation [53].

### 3.2. Analysis of Individual Phenolic Compounds

The validation of the HPLC procedure was made by evaluating linearity, the limit of detection (LOD) and quantification (LOQ), intra- and inter-day precision, recovery, and stability. In Table 2, good linearity was confirmed over the determined ranges for all analyzed phenolic compounds, with correlation coefficient (r) values significantly higher than 0.970. The mean recovery was also calculated in a satisfactory range. Moreover, the data indicate that the precision of the method was acceptable, and CV values were between 0.21% and 4.67% and 0.26% and 6.79% for intra- and inter-day variations, respectively. For the stability test, retention CV for peak area and retention time were lower than 1.8 and 0.9%, respectively. Apart from this, peak areas and retention times of phenolic compounds were sufficiently stable over 48 h. These results confirm that the analytical method had excellent resolution and sensitivity.

The contents of 10 phenolic compounds in hydromethanolic and water extracts prepared from the commercial samples of *A. membranaceus* are summarized in Table 3. In contrast to hydromethanolic extracts, CNA, RUT, and NAR were not found in water extracts, while VA, PAT, *p*CA FA, and API were not found in both extracts of *A. membranaceus*. In general, higher amounts of phenolic compounds were determined in the hydromethanolic than in the water extracts. The efficiency of phenolic extraction depends, among other factors, on the extraction method and solvent type. In our study, we prepared hydromethanolic and water extracts. Moreover, we used two different extraction methods. For hydromethanolic extracts, we performed ultrasonic extraction, and for water extracts, the plant sample was infused with boiling water. The experimental conditions could influence the obtained extraction results. In the hydromethanolic extracts, only two phenolic compounds (RUT and Q) were found in all analyzed samples, wherein Q was determined in higher concentration (89.84 µg/g DW) than RUT (53.64 µg/g DW). GA was determined in six samples, CNA in eight samples, and NAR in seven samples. In the case of water extracts, only GA and Q were determined, wherein GA was found in a higher concentration (364.83 µg/g DW) than Q (85.01 µg/g DW). In addition, the hydromethanolic extracts of samples no. 2 from Poland and 5 from USA, both in capsule form and labeled as dietary supplement, were the richest in phenolic compounds, especially in RUT, while for water extract, samples no. 2 (dietary supplement in capsule form from Poland) and 6 (cut roots from China) were the richest, especially in Q. Li et al. [51] determined VA, *p*CA, FA, RUT, and Q in *A. membranaceus* var. *mongholicus* flowers and in their ethanolic extracts found the highest concentration of RUT (1.952 mg/g) and Q (1.159 mg/g), while water extracts were richest in RUT (0.663 mg/kg) and *p*CA (0.390 mg/kg). Mahmoudi et al. [54] analyzed phenolic compounds in the seeds of *A. gombiformis*, *A. armatus*, and *A. caprinus*, and found that *A. armatus* possessed the highest level of *p*CA (39.8 µg/g DW), while RUT was determined only in *A. armatus* and *A. caprinus* seeds, at 2.94 and 5.05 µg/g DW, respectively. Moreover, the amount of API, NAR and Q were below 1 µg/g DW, while GA and PAT were found in higher concentrations.

### 3.3. Evaluation of Antioxidant Activity

It is not possible to find a single analytical method to evaluate its antioxidant capacity due to the diversity of mechanisms an antioxidant compound or mixture can exert in vivo. Consequently, the antioxidant activity of plant extracts may be quantified by different methods. In our study, we used four different methods (DPPH, FRAP, CUPRAC, and ABTS) to measure antioxidant activity as alternative tools for confirming the obtained results. It is important because an intake of a rich antioxidant diet is associated with a lower risk of chronic diseases. Thus, the measurement of the antioxidant capacity of natural products allows us to predict their antioxidant activity (higher results for the tested samples—higher antioxidant activities). The results of antioxidant activities tested for the hydromethanolic and water extracts are compiled in Table 4. Higher values of DPPH, FRAP, CUPRAC and ABTS were obtained for the infusions, 252.33 mg TAE/g, 7.02 mg Fe^2+^/g, 25.55 mg AAS/g, and 5.20 mg TAE/g, respectively, than for the hydromethanolic extracts, 196.07 mg TAE/g, 1.02 mg Fe^2+^/g, 3.16 mg AAS/g and 0.82 mg TAE/g, respectively. It could be related to higher levels of the phenolic compounds in the infusions. Moreover, hydromethanolic extracts and infusions prepared from sample no. 5 (supplement dietary in capsule form from USA) were characterized by the highest antioxidant activities among all analyzed samples, while antioxidant activities, especially FRAP values of sample no. 4 (cut roots from China) were the lowest. Literature data revealed antioxidant activity in *A. membranaceus* is scarce. Gebalski et al. [52] determined DPPH, ABTS, and FRAP values in the range from 8 to 20%, from 4 to 90% and below 10 mg TAE/g of the root of Chinese *A. membranaceus*. Otherwise, Cui et al. [55] analyzed stems and leaves of *A. membranaceus* and reported respective IC_50_ values for DPPH, ABTS, and FRAP at the levels of 0.1490 mg/mL, 0.0320 mg/mL, and 3.1939 mg/mL, respectively. The antioxidant activity values cannot be compared with those of this study due to the difference in the calculation units.

### 3.4. Evaluation of Antibacterial Activity

The extracts from *A. membranaceus* were tested for their antibacterial activity (Table 5) against two reference strains of bacteria, i.e., gram-positive *Staphylococcus aureus* and gram-negative *Escherichia coli,* using a diffusion method on a solid medium. At the tested concentration of 300 mg/mL, the inhibition zone diameters were within the range of 10 to 22 mm. This is much less effective antibacterial activity compared to ampicillin used as the reference antibiotic. For the antibiotic, growth inhibition zones of 44 mm were obtained (for *S. aureus*) at the tested concentration of 2 mg/mL. The highest activity showed a preparation 5 (dietary supplement in capsule form, from the USA) against *S. aureus* strain with an inhibition zone diameter of 18 ± 3 mm. In turn, preparations 1 (dietary supplement in cut roots form, from Poland), 4 (cut roots from China), 7 (cut roots from Russia), and 8 (in powder form, from China) showed insignificant effects against gram-positive bacteria—growth inhibition zones with a diameter of 10 ± 2 mm were obtained for these preparations. The remaining preparations did not show antibacterial activity at the tested concentrations. There were no inhibition zones in any extracts in the case of *E. coli*.

### 3.5. AChE/BChE and TYR Inhibitory Activity

Although *Astragalus* preparations are mainly recommended as dietary supplements for immune boosters and adaptogenic and anti-inflammatory agents, the research indicated its positive effects on brain health acting through various mechanisms. For instance, the root extract of *A. membranaceous* (astragalosides > 63%) was administered to 12-month-old male mice for 21 days at doses of 10, 20, and 40 mg/kg [56].

The extract caused a marked effect on memory and learning in mice via downregulating the mRNA level of caspase-3, decreasing expression of caspase-3 and cytochrome C in the hippocampus (CA1, CA3) and neocortex at doses of 20 and 40 mg/kg, which indicated the neuroprotective effect of the extract. In 7-month-aged APP/PS1 mice with memory deficit, treatment with the polysaccharide fraction of *A. membranaceous* significantly improved the cognitive ability of APP/PS1 mice, lessened apoptosis and the accumulation of Aβ, which is highly characteristic in patients with Alzheimer’s disease (AD) [57]. Relevantly, astragaloside IV was reported to prevent Aβ-induced memory shortage and hippocampal neuronal apoptosis, likely by improving the peroxisome proliferator-activated receptor γ (PPARγ)/brain-derived neurotrophic factor (BDNF) signaling pathway [58]. In a very recent study, 25 mg/kg/day of cycloastragenol orally was given for 3 weeks to Sprague-Dawley rats in an AD model induced with aluminum chloride [59]. Cycloastragenol was shown to possess a positive effect in hippocampal situations in rat brains in addition to its anti-apoptotic and anti-inflammatory activities through diminishing the expression of NF-κB, TNF-α, BAX, and caspase-3. Astragaloside was observed to repair microwave-impaired spatial learning and memory ability in rats by restoring the acetylcholine level in rat hippocampus as well as improving mitochondrial swelling and cavitation, rough endoplasmic reticulum swelling and dilation, synaptic gap disappearing, and vesicle aggregation, which was also proved by brain electroencephalogram (EEG) [60].

There have been only a few studies on the cholinesterase or TYR inhibitory effect of extracts and the single components of *A. membranaceous*. In a screening study, a methanol extract of A. membranaceous was lately tested for its cholinesterase and TYR inhibitory effects by Gebalski et al. [52]. The extract displayed very low inhibition against AChE (below 10%) in comparison to eserine (syn. physostigmine) employed as the reference, which is in complete agreement with our data on the preparations of the plant. Accordingly, its ethanolic extract was also reported to show a weak anti-AChE effect [61]. On the other hand, astragalosides II, III, and IV, when tested individually using the TLC bioautography method, possessed varying levels of AChE inhibition with their respective IC50 values of 5.9, 4.2, and 4.0 μM [62]. In our previous study, we reported a very low rate of AChE and BChE inhibition (< 10%) by astragalosides I, IV, and VI, as well as astrasieversianin II and astrasieversianin X earlier isolated from the roots of *A. melanophrurius* Boiss [63]. The root extract of *A. membranaceous* exhibited an inhibition rate below 40% against TYR [52], which is similar to our findings herein (Table 6). Among tested extracts of *A. membranaceous* samples, only two of them, e.g., sample no. 5 (dietary supplement in capsule form, from USA) and 10 (in powder form, from USA), were able to inhibit BChE with better IC_50_ values of 42.65 ± 2.5 and 32.65 ± 2.78 µg/mL (respectively) than the reference drug (galanthamine, IC_50_: 119.3 ± 1.05 µg/mL). These results could be most likely related to their phenolic phytochemical content rather than astragalosides and other saponin derivatives.

### 3.6. Correlation Analysis

In order to establish the relationship between the phenolic composition and antioxidant activity of *A. membranaceus* commercial samples, Pearson’s correlation coefficient analysis was applied. The obtained results showed 28 and 14 statistically significant correlations for hydromethanolic and water extracts of *A. membranaceus*, respectively. Among individual phenolic compounds, only one correlation was found between GA and RUT (0.67) in the hydromethanolic extracts, while no significant correlation was found in water extracts of *A. membranaceus*. The relationship between the individual phenolic compounds and the antioxidant potential revealed that GA and RUT in hydromethanolic extracts of *A. membranaceus* strongly correlated with DPPH values (0.83 and 0.69, respectively). Moreover, the correlations in the pairs of GA-ABTS and GA-CUPRAC were found to be also positive (0.69 and 0.71, respectively). This can indicate the crucial role of GA and RUT as antioxidant constituents in hydromethanolic extracts of *A. membranaceus*. In the case of TPCs, TFCs, TPAC, and the antioxidant activities in hydromethanolic extracts, their TPC, TFC, and TPACs were correlated with ABTS (0.77, 0.87, and 0.70, respectively) and CUPRAC (0.78, 0.94, and 0.77, respectively). Moreover, their TFCs and TPACs were also correlated with DPPH (0.90 and 0.76, respectively), and TFCs were correlated with FRAP (0.77). TPCs, TFCs, and TPACs calculated in the water extracts were correlated with FRAP (0.91, 0.75 and 0.86, respectively) and CUPRAC (0.97, 0.67 and 0.97, respectively). Besides, TPCs were correlated with ABTS (0.64), while TFCs with DPPH (0.85). In the case of the relationship between antioxidant activities, strong (r > 0.80) correlations between DPPH, ABTS, FRAP, and CUPRAC were observed in the hydromethanolic extracts, while in water extracts of *A. membranaceus*, FRAP and ABTS assays were strongly correlated to CUPRAC assay, 0.92 and 0.65, respectively. Positive correlations have already been reported between antioxidant activities and phenolic contents for other plants [64,65]. The results prove the importance of phenolic compounds in the antioxidant behavior of *A. membranaceus* extracts and also that they contribute significantly to the total antioxidant capacity.

### 3.7. Discrimination of Astragalus Membranaceus Commercial Samples

**Figure 1:**
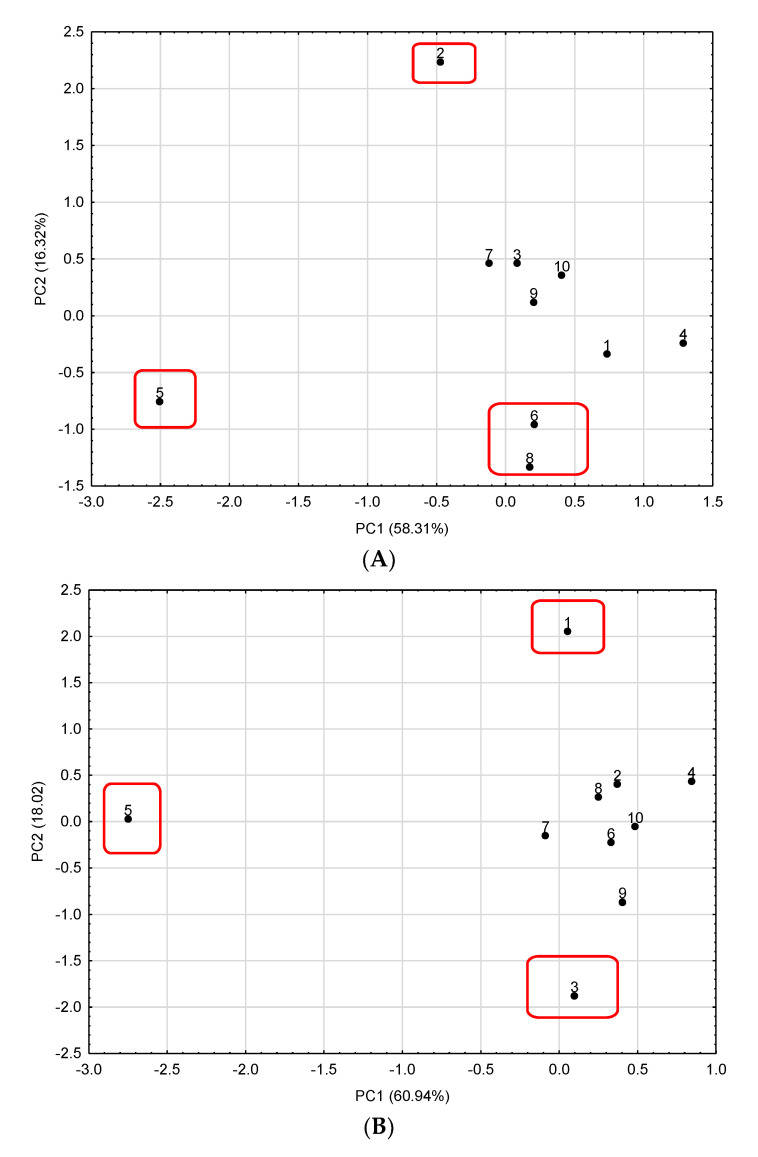
The principal component analysis score plot of the first two components for (**A**) hydromethanolic extracts and (**B**) water extracts of *A. membranaceus*.

Principal component analysis (PCA) was used to identify groups of *A. membranaceus* samples with similarities in terms of phenolic compounds and antioxidant activities. Moreover, PCA allows for the identification of some factors that describe the crucial variability in a data matrix. A graphical illustration of PCA calculation is shown in Figure 1.

For hydromethanolic extracts, the first two principal components (PCs) explained 74.63% of the total variability. PC1 was positively correlated with TPC, TFC, TPAC, RUT, GA, ABTS, DPPH, FRAP, and CUPRAC, while PC2 was positively correlated with Q and CNA. In the case of the water extracts, the first two principal components (PCs) explained 78.96% of the total variability. PC1 is positively correlated with TPC, TFC, TPAC, GA, ABTS, CUPRAC, and FRAP, while PC2 is negatively correlated with GA. Sample no. 5 (roots in loose form from the USA) can be found on the left part of the scatterplot for the hydromethanolic and water extracts. This sample is rich in phenolic compounds (Table 1 and Table 3) and has high antioxidant activities (Table 4). Moreover, for the hydromethanolic extracts, samples no. 2 (dietary supplement in capsule form from Poland), 6 (cut roots from China), and 8 (powder form from China) were well separated from the others by being on the negative region of the PC2 (samples no. 6 and 8 from China) or on the positive region (sample no. 2 as a dietary supplement from Poland) due to their significantly lower flavonoids contents. In the case of water extracts, samples no. 1 (cut roots from Poland) and 3 (cut roots from China) were also well separated from the others by being on the negative region (sample no. 3) or positive region (sample no. 1 as dietary supplement from Poland) of the PC2 due to their high content of Q. Based on obtained result for PCA analysis seems that the commercial source and form of the samples plays a negligible role in influencing the chemical composition of *A. membranaceus* extracts.

## 4. Conclusions

Hydromethanolic and water extracts prepared from *A. membranaceus* commercial samples were investigated in terms of the phenolic profile and in vitro antioxidant, antimicrobial and enzyme inhibitory (AChE, BChE, and TYR) activities. The obtained results showed that water extracts are richer in phenolic compounds and possess higher antioxidant activity than hydromethanolic extracts. Among the individual phenolic constituents, only Q was found in both extracts, while VA, PAT, *p*CA, FA and API were not detected. Both extracts prepared from sample no. 5 (roots in loose form from USA) were characterized by the highest phenolic contents and antioxidant activities, while sample no. 4 (cut roots from China) was the poorest among all analyzed samples. Regarding the antimicrobial properties of *A. membranaceus* commercial samples, samples no. 1, 4, 7, and 8 showed insignificant effects against *S. aureus*, while there were no inhibition zones in any extracts in the case of *E. coli*. Besides, *A. membranaceus* extracts showed an inhibition rate below 30% against TYR, and only two samples (no. 5 and 10) were able to inhibit BChE with better IC_50_ than the reference drug (galanthamine). Finally, correlation analysis indicated a strong positive relationship between antioxidant activities and phenolic composition. The results obtained suggest the crucial role of phenolic constituents as antioxidant agents in *A. membranaceus* extracts. In conclusion, *A. membranaceus* commercial samples, specially labeled as dietary supplements, could be an important dietary source of natural antioxidants. Moreover, the commercial source and form of the samples play a negligible role in influencing the chemical composition of *A. membranaceus* extracts. However, based on the results from this study, the products available over the counter do not ensure that they always possess these effects, and further control is needed.

## Figures and Tables

**Table 1:** Results of total phenolic (TPCs), flavonoid (TFCs) and phenolic acid (TPACs) contents of hydromethanolic and water extracts of *Astragalus membranaceus* commercial samples.

Sample No.	TPAC [µg CAE/g]	TPC [µg GAE/g]	TFC [µg QE/g]	TPAC [µg CAE/g]	TPC [mg GAE/g]	TFC [µg QE/g]
	Hydromethanolic Extracts			Water Extracts		
1	84.56 ± 2.31 ^d^	409.60 ± 0.78 ^a^	69.56 ± 0.45 ^a^	542.31 ± 2.80 ^bc^	1.33 ± 0.54 ^a^	550.70 ± 2.34 ^bc^
2	102.53 ± 0.93 ^e^	478.99 ± 1.02 ^bc^	75.04 ± 0.52 ^ab^	574.38 ± 1.56 ^ab^	1.04 ± 0.34 ^b^	735.08 ± 1.89 ^e^
3	111.38 ± 2.83 ^f^	576.81 ± 1.48 ^d^	71.22 ± 0.54 ^a^	679.12 ± 2.81 ^d^	1.23 ± 0.25 ^ac^	849.80 ± 2.33 ^f^
4	59.10 ± 1.58 ^a^	325.31 ± 1.19 ^e^	59.32 ± 0.56 ^a^	496.21 ± 1.65 ^c^	0.96 ± 0.09 ^b^	414.53 ± 2.82 ^d^
5	143.79 ± 3.56 ^g^	577.70 ± 1.98 ^d^	170.39 ± 0.55 ^b^	2139.97 ± 1.26 ^e^	3.36 ± 0.53 ^d^	1396.14 ± 2.18 ^g^
6	68.55 ± 3.89 ^bc^	443.92 ± 1.65 ^ab^	65.94 ± 0.47 ^a^	727.27 ± 1.48 ^d^	1.09 ± 0.64 ^bc^	487.38 ± 2.02 ^a^
7	57.80 ± 2.23 ^a^	480.73 ± 1.31 ^bc^	68.56 ± 0.81 ^a^	601.26 ± 2.44 ^a^	1.24 ± 0.12 ^ac^	501.65 ± 2.50 ^ab^
8	66.81 ± 1.36 ^bc^	445.49 ± 1.20 ^ab^	66.49 ± 0.49 ^a^	591.19 ± 3.96 ^ab^	1.34 ± 0.23 ^a^	561.81 ± 1.39 ^c^
9	72.47 ± 1.14 ^c^	507.07 ± 0.83 ^c^	67.66 ± 0.50 ^a^	570.80 ± 1.27 ^ab^	1.31 ± 0.31 ^a^	513.54 ± 1.53 ^abc^
10	64.76 ± 2.13 ^ab^	412.56 ± 2.6 ^a^	66.98 ± 1.0 ^a^	612.65 ± 3.91 ^a^	1.11 ± 0.10 ^bc^	522.80 ± 2.75 ^abc^

The results in the same column followed by the same letters do not significantly differ by Tukey’s HSD test (*p* < 0.05).

**Table 2:** Validation parameters of the calibration curves for analytes quantified in this study (n = 3).

Analytes	Regression Equation	Linearity (µg/mL)	R^2^	LOD (µg/mL)	LOQ (µg/mL)	Recovery (%)
GA	y = 20,025x − 29,083	56–280	0.983	3.52	10.02	96.57
PAT	y = 39,674x + 19,375	55.5–276	0.983	2.46	7.04	96.49
CNA	y = 35,424x + 14,534	48.4–242	0.987	2.65	7.66	95.79
VA	y = 30,176x + 56,284	53.6–268	0.981	2.65	7.13	103.97
FA	y = 22,525x + 17,955	52–260	0.980	3.54	9.68	91.86
*p*CA	y = 98,491x + 17,039	55.6–278	0.979	4.54	12.53	91.89
API	y = 34,257x + 14,385	51.4–260	0.985	3.12	8.47	95.78
NAR	y = 42,541x + 51,683	57.6–288	0.998	2.38	7.14	93.88
RUT	y = 13,223x + 29,642	46.4–234	0.970	3.01	8.56	95.57
Q	y = 17,992x − 35,440	42–210	0.979	5.68	12.44	96.43

y is the peak area; x refers to the concentration of compounds (µg/mL).

**Table 3:** The results of individual phenolic compounds in *A. membranaceus* commercial samples (mean ± standard deviation).

	GA	VA	PAT	*p*CA	FA	CNA	RUT	Q	API	NAR
Hydromethanolic extracts (μg/g)										
1	39.92 ± 4.10 ^a^	ND	ND	ND	ND	ND	9.18 ± 1.76 ^a^	43.04 ± 5.20 ^ab^	ND	ND
2	ND	ND	ND	ND	ND	69.67 ± 3.57 ^f^	114.88 ± 3.29 ^g^	228.34 ± 6.16 ^g^	ND	ND
3	ND	ND	ND	ND	ND	31.29 ± 1.14 ^bc^	12.45 ± 2.03 ^a^	45.63 ± 1.49 ^b^	ND	ND
4	73.32 ± 4.37 ^b^	ND	ND	ND	ND	ND	13.60 ± 1.77 ^a^	80.72 ± 5.13 ^c^	ND	28.55 ± 3.79 ^a^
5	705.80 ± 9.35 ^f^	ND	ND	ND	ND	17.17 ± 0.80 ^a^	140.74 ± 2.77 ^h^	83.57 ± 4.43 ^c^	ND	117.08 ± 6.33 ^b^
6	376.60 ± 12.09 ^e^	ND	ND	ND	ND	35.40 ± 0.24 ^c^	76.88 ± 1.97 ^f^	39.86 ± 3.43 ^a^	ND	828.32 ± 11.45 ^g^
7	310.10 ± 8.69 ^d^	ND	ND	ND	ND	28.43 ± 0.79 ^a^	68.37 ± 3.72 ^e^	187.63 ± 4.15 ^f^	ND	223.62 ± 6.23 ^e^
8	263.78 ± 5.61 ^c^	ND	ND	ND	ND	4.05 ± 0.47 ^d^	33.89 ± 2.28 ^c^	44.53 ± 1.49 ^ab^	ND	689.52 ± 9.60 ^f^
9	ND	ND	ND	ND	ND	17.68 ± 1.37 ^a^	22.82 ± 1.74 ^b^	93.37 ± 3.44 ^e^	ND	189.65 ± 6.99 ^c^
10	ND	ND	ND	ND	ND	51.67 ± 4.78 ^e^	43.56 ± 3.75 ^d^	51.70 ± 4.11 ^d^	ND	210.61 ± 5.77 ^d^
Water extracts (μg/g)										
1	315.34 ± 6.69 ^a^	ND	ND	ND	ND	ND	ND	163.95 ± 9.94 ^f^	ND	ND
2	363.62 ± 4.70 ^f^	ND	ND	ND	ND	ND	ND	130.38 ± 7.85 ^e^	ND	ND
3	481.40 ± 5.49 ^g^	ND	ND	ND	ND	ND	ND	105.64 ± 3.52 ^d^	ND	ND
4	317.59 ± 5.25 ^ab^	ND	ND	ND	ND	ND	ND	76.63 ± 2.48 ^b^	ND	ND
5	353.83 ± 12.75 ^c^	ND	ND	ND	ND	ND	ND	77.38 ± 1.29 ^b^	ND	ND
6	404.64 ± 9.10 ^d^	ND	ND	ND	ND	ND	ND	57.86 ± 0.68 ^a^	ND	ND
7	352.80 ± 4.12 ^c^	ND	ND	ND	ND	ND	ND	58.38 ± 0.75 ^a^	ND	ND
8	333.20 ± 2.59 ^e^	ND	ND	ND	ND	ND	ND	56.88 ± 0.26 ^a^	ND	ND
9	402.63 ± 6.22 ^d^	ND	ND	ND	ND	ND	ND	58.75 ± 3.73 ^a^	ND	ND
10	323.23 ± 5.99 ^b^	ND	ND	ND	ND	ND	ND	64.23 ± 3.97 ^c^	ND	ND

The results in the same column followed by the same letters do not significantly differ by Tukey’s HSD test (*p* < 0.05); ND: not detectable.

**Table 4:** The results of antioxidant activities of *Astragalus membranaceus* commercial samples.

Sample No.	DPPH (mg TEA/g)	FRAP (mgFe^2+^/g)	CUPRAC (mg ASA/g)	ABTS (mg TEA/g)
Hydromethanolic extracts				
1	159.47 ± 2.85 ^ab^	0.74 ± 0.02 ^e^	2.70 ± 0.05 ^b^	0.62 ± 0.02 ^c^
2	159.71 ± 1.91 ^ab^	1.11 ± 0.04 ^ab^	3.27 ± 0.14 ^a^	0.87 ± 0.01 ^b^
3	167.77 ± 1.85 ^ac^	0.63 ± 0.01 ^c^	3.17 ± 0.11 ^a^	0.81 ± 0.02 ^ae^
4	126.91 ± 6.58 ^d^	0.56 ± 0.06 ^c^	2.22 ± 0.10 ^d^	0.62 ± 0.03^c^
5	272.96 ± 13.87 ^e^	1.76 ± 0.02 ^f^	4.67 ± 0.07 ^e^	1.17 ± 0.02 ^f^
6	167.97 ± 4.12 ^ac^	0.98 ± 0.02 ^d^	2.85 ± 0.09 ^bc^	0.75 ± 0.02 ^d^
7	179.24 ± 7.49 ^c^	1.10 ± 0.03 ^ab^	3.21 ± 0.14 ^a^	0.86 ± 0.03 ^ab^
8	184.53 ± 1.57 ^c^	1.13 ± 0.02 ^a^	3.22 ± 0.09 ^a^	0.82 ± 0.01 ^ab^
9	146.08 ± 4.93 ^b^	1.15 ± 0.01 ^a^	3.10 ± 0.14 ^ac^	0.83 ± 0.04 ^ab^
10	157.87 ± 2.65 ^ab^	1.05 ± 0.04 ^bd^	2.99 ± 0.97 ^abc^	0.76 ± 0.08 ^de^
Water extracts				
1	139.89 ± 6.37 ^e^	7.92 ± 0.21 ^a^	25.90 ± 0.79 ^b^	5.48 ± 0.19 ^de^
2	205.50 ± 4.58 ^ab^	6.35 ± 0.16 ^a^	22.51 ± 0.85 ^ab^	4.99 ± 0.17 ^abc^
3	365.48 ± 7.12 ^d^	5.93 ± 0.19 ^a^	20.99 ± 1.09 ^a^	4.96 ± 0.21 ^abc^
4	176.38 ± 5.82 ^a^	5.86 ± 0.13 ^a^	21.32 ± 1.18 ^a^	4.66 ± 0.16 ^a^
5	389.64 ± 6.64 ^d^	10.29 ± 0.85 ^b^	47.91 ± 3.10 ^c^	5.76 ± 0.76 ^e^
6	178.80 ± 2.82 ^a^	6.46 ± 0.13 ^a^	24.10 ± 0.53 ^ab^	5.26 ± 0.16 ^cd^
7	329.22 ± 5.73 ^f^	7.35 ± 0.17 ^a^	23.30 ± 1.69 ^ab^	5.59 ± 0.17 ^de^
8	216.70 ± 2.88 ^b^	6.67 ± 0.10 ^a^	22.18 ± 0.88 ^a^	5.23 ± 0.15 ^bcd^
9	269.33 ± 8.16 ^c^	6.36 ± 0.11 ^a^	21.75 ± 1.20 ^a^	4.86 ± 0.08 ^ab^
10	254.52 ± 3.67 ^c^	6.02 ± 0.87 ^a^	23.88 ± 2.08 ^ab^	4.76 ± 1.09 ^a^

The results in the same column followed by the same letters do not significantly differ by Tukey’s HSD test (*p* < 0.05).

**Table 5:** Antimicrobial activity of water extracts of *A. membranaceus* commercial samples against tested bacterial strains.

Sample No.	*Stapphylococcus aureus* ATCC6538	*Escherichia coli* ATCC8739
1	10/10	nz
2	0/0	nz
3	0/0	nz
4	10/10	nz
5	22/15	nz
6	0/0	nz
7	12/10	nz
8	12/10	nz
9	13/10	nz
10	0/0	nz

Diameter of the bacterial growth inhibition zone in millimeters. nz—non-zone.

**Table 6:** BChE and TYR inhibitory activity of *A. membranaceus* commercial samples.

Sample No.	BChE Inhibition (Inhibition % ± S.D.^a^) 200 µg/mL ^b^	TYR Inhibition (Inhibition % ± S.D.^a^) 100 µg/mL^b^
1	16.22 ± 0.02	5.22 ± 0.12
2	27.31 ± 0.12	8.13 ± 0.11
3	31.22 ± 0.17	11.02 ± 0.09
4	47.17 ± 1.13	19.27 ± 0.12
5	55.17 ± 2.12 (IC_50_: 42.65 ± 2.5 µg/mL)	27.16 ± 1.3
6	42.98 ± 0.15	7.38 ± 0.83
7	47.15 ± 0.22	12.01 ± 0.43
8	13.22 ± 0.15	3.12 ± 0.11
9	48.67 ± 0.14	6.12 ± 0.01
10	61.14 ± 1.02 (IC_50_: 32.65 ± 2.78 µg/mL)	16.12 ± 0.12
References	66.91 ± 2.32 ^c^ (IC_50_: 119.3 ± 1.05 µg/mL)	76.58 ± 0.85 ^d^ (IC_50_: 52.42 ± 2.67 µg/mL)

^a^ Standard deviation (n: 3), ^b^ Final concentration, ^c^ Galanthamine hydrobromide (100 µg/mL), ^d^ α-Kojic acid (200 μg/mL).

## Data Availability

Data is available upon request.
